# Heterogeneous Glycation of Cancellous Bone and Its Association with Bone Quality and Fragility

**DOI:** 10.1371/journal.pone.0035047

**Published:** 2012-04-13

**Authors:** Lamya Karim, Deepak Vashishth

**Affiliations:** Center for Biotechnology and Interdisciplinary Studies, Department of Biomedical Engineering, Rensselaer Polytechnic Institute, Troy, New York, United States of America; Ohio State University, United States of America

## Abstract

Non-enzymatic glycation (NEG) and enzymatic biochemical processes create crosslinks that modify the extracellular matrix (ECM) and affect the turnover of bone tissue. Because NEG affects turnover and turnover at the local level affects microarchitecture and formation and removal of microdamage, we hypothesized that NEG in cancellous bone is heterogeneous and accounts partly for the contribution of microarchitecture and microdamage on bone fragility. Human trabecular bone cores from 23 donors were subjected to compression tests. Mechanically tested cores as well as an additional 19 cores were stained with lead-uranyl acetate and imaged to determine microarchitecture and measure microdamage. Post-yield mechanical properties were measured and damaged trabeculae were extracted from a subset of specimens and characterized for the morphology of induced microdamage. Tested specimens and extracted trabeculae were quantified for enzymatic and non-enzymatic crosslink content using a colorimetric assay and Ultra-high Performance Liquid Chromatography (UPLC). Results show that an increase in enzymatic crosslinks was beneficial for bone where they were associated with increased toughness and decreased microdamage. Conversely, bone with increased NEG required less strain to reach failure and were less tough. NEG heterogeneously modified trabecular microarchitecture where high amounts of NEG crosslinks were found in trabecular rods and with the mechanically deleterious form of microdamage (linear microcracks). The extent of NEG in tibial cancellous bone was the dominant predictor of bone fragility and was associated with changes in microarchitecture and microdamage.

## Introduction

Age-related bone fracture is a major health problem that can result in disability and substantial medical care costs [1]. Low bone mass or bone mineral density (BMD) is considered the key factor in determining loss of bone strength and increased fracture risk [2], [3]. However, studies demonstrate that decreased bone mass alone is not sufficient to cause a fracture [4]–[6]. Therefore, other factors such as changes in bone quality [7] may also be key determinants of fracture risk. Bone quality can be altered by several features including changes in the extracellular matrix (ECM) and microarchitecture, which interact under applied loading to influence microdamage formation that can contribute to bone's mechanical properties [7], [8].

Bone's ECM components undergo numerous biochemical changes with aging. Any changes in these constituents can detrimentally alter the mechanical behavior of bone and explain the increased fracture incidence in the aging population [5]. In particular, type I collagen is susceptible to both enzymatic and non-enzymatic biochemical changes [9]. Both pathways yield crosslinks, but they affect bone's mechanical properties differently. Enzymatic processes produce pyridinium crosslinks (e.g. pyridinoline [Pyd], deoxypyridinoline [Dpd]) [10], [11], which are necessary for the maturation and mechanical integrity of collagen. On the other hand, non-enzymatic glycation creates advanced glycation end products (AGEs) [9], [12], [13]. Glycation-induced crosslinks are inversely related to bone toughness [14] and specific AGEs such as pentosidine explain up to 23% of the variation in bone fracture toughness [15]. However, since pentosidine is only a single component of the total fluorescent AGE content in bone, pentosidine measurement may not be fully illustrative of the overall influence of glycation on bone fragility [16], [17]. AGE accumulation has been shown to reduce osteoclastic bone resorption and lead to reduced turnover [18]. Because turnover affects formation and removal of microdamage and the local microarchitecture, AGEs may act through reduced turnover and influence other measures of bone quality associated with bone fragility.

To date, two studies have been conducted on the effect of crosslinks on trabecular microarchitecture [19], [20] and only a single study has explored the effects of non-enzymatic glycation on microdamage formation [21]. These three components of bone quality may interact with each other and determine the magnitude of bone fragility, but their relative influence on each other is currently unknown. For example, both microarchitecture and bone matrix composition may interact with applied loading to influence microdamage formation and determine mechanical properties, which illustrate bone's ability to resist fracture [22]–[24].

More importantly, it has been recently shown that the presence of rod-like trabeculae, characteristic of osteoporotic bone, makes cancellous bone more susceptible to form harmful microdamage [25]–[27]. However, no information is available on the extent of non-enzymatic glycation and microdamage formation within single trabeculae. This information can help determine if NEG in cancellous bone is heterogeneous and explain the matrix-based mechanism through which cancellous bone transitions into a fragile and weak structure.

Thus, the goal of this study was to determine whether the trabecular microarchitecture of cancellous bone is heterogeneously modified by NEG, and whether AGEs are associated with changes in microarchitecture and microdamage and predict the fracture properties of cancellous bone. To pursue this goal, cancellous bone specimens from adult human tibiae were subjected to compression tests and then quantified for microarchitecture, microdamage, and the extent of non-enzymatic glycation. Extracted trabeculae were characterized for microdamage and quantified for AGEs.

## Methods

### Specimen Collection

42 cancellous bone cores (7.5 mm diameter, 10.5 mm length) were obtained from tibial plateaus of male (n = 24) and female (n = 18) human donors ranging from age 18 to 97 (average 59.3±22.1). None of the donors were diagnosed with osteoarthritis, and they were also certified to be free of bone metabolic diseases, HIV, and hepatitis B (National Disease Research Interchange and International Institute for the Advancement of Medicine). All specimens were stored in −80°C in saline soaked gauze until use.

### Microarchitecture and Microdamage Analysis

Each specimen was scanned using micro-computed tomography (Scanco Medical AG) at 17.5 micron voxel resolution to obtain the following parameters: volumetric bone mineral density (BMD), bone volume fraction (BV/TV), connectivity density (Conn.D), structure model index (SMI), trabecular number (Tb.N), trabecular thickness (Tb.Th), and trabecular separation (Tb.Sp). Thresholds used to evaluate specimens were determined empirically based on 2D evaluations of a subset of samples.

Mechanical data was collected for a subset of the bone cores chosen at random (male n = 11, female n = 12, age range = 18–97, average 60.1±23.1). Uniaxial unconfined compression tests to post-yield (1.1%) apparent-level strain were performed using established protocols [28]–[30]. The mechanical testing was conducted at 37°C in physiological saline using a Bionix 858 servo-hydraulic testing system (MTS) with TestStar II software. An extensometer (MTS) was used for strain data collection. Stress-strain data were used to determine several mechanical parameters including elastic modulus, yield point (using the 0.2% offset method), ultimate point (determined as the point of maximum load), post-yield strain (determined as the difference between ultimate strain and yield strain), post-yield strain energy, and toughness [28], [29], [31].

All bone cores were stained using a recently developed technique allowing for the non-invasive measurement of microdamage through micro-computed tomography. The cores were cleaned of excess blood, fat, and marrow, and stained in a lead-uranyl acetate solution containing an equal mixture of 20% lead acetate in 70% acetone and 8% uranyl acetate in 70% acetone for 2 weeks, followed by a 1 week submersion in 1% ammonium sulfide in acetone [21], [32], [33].

After staining, the cores were scanned via micro-computed tomography with the same parameters used previously. Microdamage was characterized in a central 8 mm^3^ cubic region. The ratio of damaged volume to bone volume was calculated to quantify microdamage [21]. This same ratio was also determined in the region of minimum BV/TV since that region offers a more accurate prediction of bone fragility compared to overall BV/TV [34]. The minimum BV/TV region was obtained by dividing the 8 mm^3^ cubic region into four equally sized volumes and then selecting the volume with the smallest BV/TV value [27].

19 individual damaged trabeculae were extracted from a subset of specimens chosen randomly using a random number generator function in Microsoft Excel (male n = 10, female n = 9). These specimens ranged in age from 18 to 97 (average 53.8±25.1). Each trabecula was imaged via microCT using the above-specified parameters. The ratio of damaged surface area to damaged bone volume was calculated to characterize the morphology of microdamage where higher values indicate more crack-like microdamage and lower values indicate more diffuse-like microdamage [21], [27].

### Specimen Preparation for Crosslink Analysis of Bone Cores and Damaged Trabeculae

Individual trabeculae, analyzed for microdamage, were lyophilized overnight using a freeze dry system (Labconco). They were then hydrolyzed in 6N hydrochloric acid for 20 hours at 110°C. Hydrosylates were centrifuged for 30 minutes at 13000 rpm at 4°C to remove any debris. Centrifuged hydrosylates were stored at −80°C in complete darkness until use. A bone slice (7.5 mm diameter, 1 mm thickness) obtained from each of the 42 bone cores, was also hydrolyzed and stored in the same fashion. Collagen quality and purity obtained through this method of hydrolysis has been previously confirmed in our laboratory via sodium dodecyl sulfate-polyacrylamide gel electrophoresis [35]. Also, work done in our laboratory has verified that there is no difference in non-enzymatic glycation content measured in bone with and without lead-uranyl acetate stain.

### Measurement of Total Fluorescent AGEs

The fluorescence was measured for quinine standards (stock: 10 µg/mL quinine per 0.1 N sulfuric acid) and hydrosylates from individual trabeculae and bone slices at 360/460 nm excitation/emission using an Infinite 200 microplate reader (Tecan). Chloramine-T, 3.15 M perchloric acid, and p-dimethylaminobenzaldehyde solutions were all made immediately before use. Chloramine-T was added to hydroxyproline standards (stock: 2000 µg/mL L-hydroxyproline per 0.001 N HCl) and sample hydrosylates. The resulting solution was then incubated at room temperature for 20 minutes to oxidize hydroxyproline. To quench chloramine-T, 3.15 M perchloric acid was added and incubated for 5 minutes at room temperature. Finally, the p-dimethylaminobenzaldehyde solution was added and incubated for 20 minutes at 60°C. All standards and samples were cooled in darkness for 5 minutes. The absorbance was measured at 570 nm using a microplate reader. Collagen content was calculated based on the hydroxyproline quantity measured [36]. Total fluorescent AGEs were quantified in terms of unit quinine per unit collagen. Individual trabeculae were quantified for total fluorescent AGEs only, while bone slices from whole bone cores were quantified for both total fluorescent AGEs as well as specific collagen crosslinks as described in the next section.

### Measurement of Pyd, Dpd, and Pentosidine

**Figure 1:**
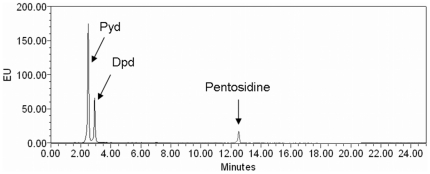
Schematic of UPLC chromatogram. Eluted peak areas were used for quantification of Pyd, Dpd, and pentosidine.

The hydrosylate from each bone slice was divided in two parts and each part was used to measure collagen content or crosslinks by Ultra-high Performance Liquid Chromatography (UPLC, Waters). A hydroxyproline kit (BioRad) was used for hydroxyproline quantification. A Pyd/Dpd calibrator (Quidel) and pentosidine stock (kindly donated by Dr. Vincent Monnier's group from Case Western Reserve University) were used as standards for Pyd, Dpd, and pentosidine quantification using recently developed methods [35]. This technique has been validated where the coefficient of variation for the intra-assay and inter-assay for Pyd, Dpd, and pentosidine were all ≤2% [35]. All separations were performed for standards and each specimen hydrosylate (Figure 1). Separations for hydroxyproline quantification were completed at 60°C with a flow rate of 0.5 mL/min (detector: 471 nm). For crosslink quantification, separations were achieved at 40°C with a flow rate of 0.667 mL/min (Pyd and Dpd at 297/395 nm excitation/emission, pentosidine at 335/385 nm excitation/emission). Collagen content was calculated based on the measured hydroxyproline content [36]. All crosslink quantities were normalized to the amount of collagen.

### Statistical Analysis

Outliers were determined as points beyond two standard deviations of the mean and were removed from analysis. Non-parametric statistical tests were used for all measures taken in whole bone specimens because Kolmogorov-Smirnov normality tests determined that several data sets were not normally distributed. Spearman correlations were run between enzymatic crosslink content (Pyd/Dpd), measures of non-enzymatic glycation (AGEs and pentosidine), microarchitecture, and microdamage for all specimens. These measures were also tested for correlation with mechanical parameters measured in the subset of 23 specimens. Forward stepwise regression tests were conducted for mechanical properties in order to determine significantly influential variables. Since data sets were normally distributed for individual trabeculae, Pearson correlations were run between SMI and the ratio of damaged surface area to damaged volume as well as between SMI and total fluorescent AGE content for each trabecula. The power to detect correlations was approximately 87%. All statistical tests were performed using SigmaStat version 2.03 (SPSS).

## Results

**Figure 2:**
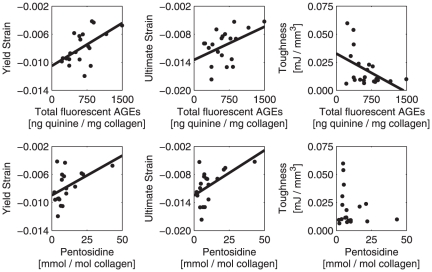
Mechanical properties versus NEG crosslinks. Graphical data for several mechanical parameters versus total fluorescent AGEs (top) and pentosidine (bottom) are shown.

Pentosidine content and total fluorescent AGEs were positively correlated (r = 0.40, p<0.05) at the level of bone cores. However, they both showed different magnitudes of association with microarchitecture, microdamage, and mechanical properties (Table 1). Highly glycated samples had more trabecular rods than plates (higher SMI) and also contained more microdamage. Mechanical measures related to bone's fracture resistance (yield strain, ultimate strain, and toughness) deteriorated in samples with high levels of non-enzymatic glycation (Table 1, Figure 2).

**Table 1:** Relationships between crosslink content and mechanical properties, microarchitecture, and microdamage content.

		Total fluorescent AGEs	Pentosidine	Pyd/Dpd
		[ng Quinine/mg collagen]	[mmol/mol collagen]	[mg/µmol]
**Mechanical properties**	**Elastic modulus [N/mm^2^]**	0.13 (NS)	0.13 (NS)	0.29 (NS)
	**Yield stress [N/mm2]**	0.01 (NS)	−0.17 (NS)	0.28 (NS)
	**Yield strain**	**0.54 (<0.01)**	**0.46 (<0.05)**	−0.45 (NS)
	**Ultimate stress [N/mm2]**	−0.03 (NS)	−0.21 (NS)	0.32 (NS)
	**Ultimate strain**	**0.38 ( = 0.09)**	**0.44 (<0.05)**	−0.36 (NS)
	**Toughness [mJ/mm^3^]**	**−0.39 ( = 0.08)**	−0.25 (NS)	**0.49 ( = 0.06)**
**Microarchitecture**	**BV/TV**	0.06 (NS)	−0.14 (NS)	**0.38 (<0.05)**
	**Conn/D [1/mm^3^]**	0.21 (NS)	−0.02 (NS)	**0.43 (<0.05)**
	**SMI**	**0.54 (<0.01)**	**0.38 (<0.05)**	−0.29 ( = 0.11)
	**Tb.N [1/mm]**	0.29 (NS)	−0.11 (NS)	**0.57 (<0.01)**
	**Tb.Th [mm]**	0.03 (NS)	0.09 (NS)	**0.35 (<0.05)**
	**Tb.Sp [mm]**	0.27 (NS)	0.22 (NS)	**−0.56 (<0.01)**
**Microdamage content**	**Global level**	0.26 (NS)	**0.35 ( = 0.054)**	**−0.39 (<0.05)**
	**Region of minimum BV/TV**	**0.43 (<0.05)**	**0.55 (<0.01)**	−0.20 (NS)

Data is presented as the Spearman correlation coefficient with the p-value in parentheses. NS = not significant. Variables showing significance (p<0.05 but <0.10) are shown in bold.

**Figure 3:**
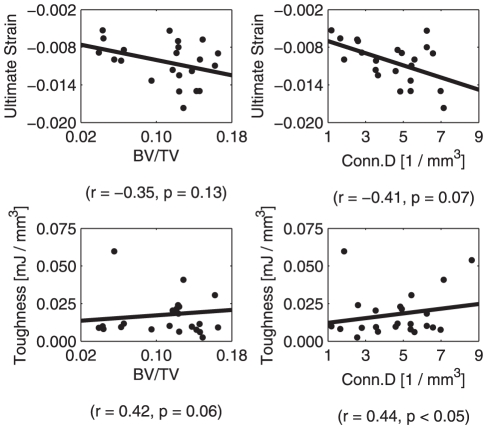
Mechanical properties versus microarchitecture. Several microarchitectural properties were correlated with cancellous bone's mechanical parameters.

In addition their relationships with the extent of non-enzymatic glycation, microarchitecture and microdamage were also associated with each other. We found that with increases in microdamage quantity, there was deterioration in microarchitecture on both the global level and region of minimum BV/TV (Table 2). Also, although several measures of microarchitecture, namely BV/TV and Conn.D, had some association with bone's mechanical properties (Figure 3), microdamage showed a trend towards significance with yield and ultimate strain (Table 2).

**Table 2:** Relationships between microdamage content, mechanical properties, and microarchitecture.

		Microdamage content	Microdamage content
		Global level	Region of minimum BV/TV
**Mechanical properties**	**Elastic modulus [N/mm^2^]**	−0.09 (NS)	0.00 (NS)
	**Yield stress [N/mm2]**	−0.37 (NS)	−0.34 (NS)
	**Yield strain**	**0.41 ( = 0.08)**	0.37 (NS)
	**Ultimate stress [N/mm2]**	−0.39 (NS)	**−0.39 ( = 0.09)**
	**Ultimate strain**	0.38 (NS)	0.29 (NS)
	**Toughness [mJ/mm^3^]**	−0.06 (NS)	0.10 (NS)
**Microarchitecture**	**BV/TV**	**−0.37 (<0.05)**	**−0.33 (<0.05)**
	**Conn/D [1/mm^3^]**	**−0.34 (<0.05)**	−0.19 (NS)
	**SMI**	**0.30 ( = 0.08)**	**0.38 (<0.05)**
	**Tb.N [1/mm]**	**−0.43 (<0.01)**	**−0.31 ( = 0.07)**
	**Tb.Th [mm]**	−0.16 (NS)	0.00 (NS)
	**Tb.Sp [mm]**	**0.50 (<0.01)**	**0.42 (<0.05)**

Data is presented as the Spearman correlation coefficient with the p-value in parentheses. NS = not significant. Variables showing significance (p<0.05 but <0.10) are shown in bold.

At the level of individual trabeculae, there were relationships between microarchitecture (SMI) and microdamage content. An increase in the amount of crack-like microdamage (high ratio of damaged surface area to damaged volume) was associated with an increase in SMI (i.e. increase in the relative proportion of trabecular rods over plates) (r = 0.48, p<0.05). Trabeculae containing crack-like microdamage were more glycated than trabeculae containing diffuse-like damage (r = 0.49, p<0.05).

Two other major variables, donor age and BMD, were associated with several properties but did not directly correlate with mechanical parameters. Pentosidine (r = 0.44, p<0.01), but not total fluorescent AGE content (r = 0.20, p = 0.27), increased with age. Also, microdamage content at a global level increased (r = 0.31, p = 0.06) increased with age. Age had no significant relationship with microarchitecture. On the other hand, BMD was positively correlated with BV/TV (r = 0.26, p = 0.10) and Tb.Th (r = 0.42, p<0.01). However, BMD had no relationship with microdamage or non-enzymatic glycation content.

There was no relationship between enzymatic crosslinks and age or BMD. Pyd/Dpd was negatively associated with pentosidine (r = −0.27, p = 0.054), but not with total fluorescent AGEs. Additionally, increased Pyd/Dpd was associated with increased bone toughness and decreased microdamage content (Table 1). Pyd/Dpd was also associated with several microarchitectural properties (Table 1).

Forward stepwise regression tests to predict yield strain (p<0.05), ultimate strain (p<0.05), and toughness (p<0.05) as dependent variables using all significant properties as independent variables showed that, regardless of the numerous existing interrelationships, the extent of non-enzymatic glycation (total fluorescent AGEs or pentosidine) was the only significant predictive variable of mechanical properties.

## Discussion

Non-enzymatic glycation has been shown to deteriorate bone's mechanical parameters, but its distribution within cancellous bone and the extent of its influence on fracture in comparison to other changes in bone quality remains unclear [14], [28], [37], [38]. Our goal was to determine if microarchitecture of cancellous bone is heterogeneously modified by NEG, and if AGEs predict the fracture properties of cancellous bone and correlate with changes in microarchitecture and microdamage.

In contrast to AGEs, pentosidine is more commonly used as a marker of NEG in bone [16], [28], [37]. However, it is not known whether measurement of pentosidine is adequate to illustrate the effects of non-enzymatic glycation on the mechanical integrity of bone [15]. Our results show that pentosidine explained 40% of the variance in bulk fluorescent AGE content and comprises only a portion of total fluorescent AGEs. Unlike age-dependence of pentosidine concentration [15], [39], our data indicated that total fluorescent AGEs in human tibial cancellous bone do not follow this trend and may therefore, contribute differently to the overall influence of non-enzymatic glycation. Moreover, unlike pentosidine, our data illustrated that AGE content predicted bone toughness. Thus, pentosidine alone does not represent the collective effects of all AGEs on bone fracture.

To date, only two studies have investigated the effect of crosslinking on trabecular microarchitecture. Microarchitecture is known to interact with applied loading at multiple length scales [20], [23] and form in vivo microdamage of distinct morphologies, which reflect the quality and fracture resistance of bone matrix [40]. However, one of these two studies was limited to quantification of enzymatic crosslinks while using 2D histomorphometric methods for microarchitectural imaging [19]. The other more recent work included measurement of non-enzymatic crosslinks and used 3D microCT imaging methods; however, it measured only pentosidine [20].

Out of all microarchitectural variables, our study found that increased glycation showed a strong positive correlation only with SMI. SMI illustrates the prevalence of rods and plates in a trabecular network where an increase in SMI illustrates a transition from plate-like to rod-like trabecular structure. AGEs may cause an alteration in turnover and, as a result, affect the microarchitecture. In particular, the inside of trabeculae is composed of older bone tissue that is sandwiched between trabecular packets. Working with cortical bone, we have demonstrated that older bone tissue contains higher AGE levels including pentosidine in comparison to younger bone tissue [41]. This evidence, combined with previous results showing a negative relationship between AGEs and in vitro bone resorption by osteoclasts [18], and between AGEs and bone turnover [37], strongly suggests that rods would contain more AGEs than plates, as was found in our study. Therefore, non-enzymatic glycation may act via the reduced propensity of osteoclastic resorption to retain fragile trabecular rods in the structural network.

**Figure 4:**
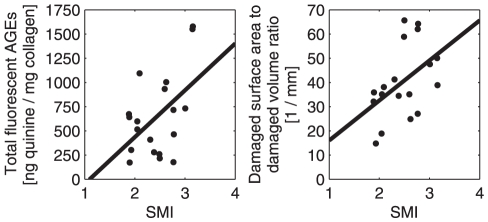
NEG influences microdamage morphology. (Left) Higher NEG levels exist in rod-like trabeculae compared to plate-like trabeculae. (Right) Also, rod-like trabeculae are prone to producing more crack-like microdamage.

Since the accumulation of AGEs increases bone fragility, the high glycation level in rod-like trabeculae may further weaken the cylindrical structures, which are already prone to bending and buckling [42]. Consistent with this notion, we found that the rod-like trabeculae are indeed more prone to producing crack-like microdamage (Figure 4), which may further contribute to the failure of trabecular rods than plates.

A recent study established that in vitro induced non-enzymatic glycation affects mechanical properties along with alterations in microdamage [21], but this study was done in a single donor. To improve on this previous work, our project explored in vivo glycation in 42 donors with a wide age and BV/TV range and investigated the relationship of non-enzymatic glycation with changes in microdamage and microarchitecture. Similar to previous findings, we found that rod-like trabeculae in a weakened microstructure are conducive to increased formation of microdamage [21], and that glycation altered the extent of accumulated microdamage in bone.

AGEs, microarchitecture, and microdamage are therefore all interrelated to bone's mechanical properties, and show relationships with yield strain, ultimate strain, and toughness in our study. Furthermore, measures of non-enzymatic glycation are also correlated to microarchitectural variables, microdamage content, and mechanical properties. Due to the host of interrelated factors, it was necessary to establish the leading variables that influence mechanical properties. Although microdamage was associated with microarchitecture, and microarchitecture was directly correlated with mechanical parameters, our forward stepwise regression analysis indicated that the extent of glycation was the only dominant predictor. Unlike previous results on cancellous bone [2], [43], we did not find correlations between microdamage content and mechanical properties in cancellous bone. These differences between our and previous studies may indicate that the effect of bone quality measures including microdamage may be site specific, and microdamage may have a greater influence on the fracture of low density vertebral bone. Further work is necessary to explain such differences.

Measurement of enzymatic crosslinks is critical because they are also determinants of bone quality [44], [45] and may positively affect bone's mechanical strength [46]. In particular, the Pyd/Dpd ratio is illustrative of collagen maturity since both of these crosslinks provide the fibers with strength and stability [47], [48]. A previous study, in which the relationship between enzymatic crosslinks and bone strength was investigated, showed that high Pyd/Dpd bone was stronger than low Pyd/Dpd bone [49]. Here, we found that high Pyd/Dpd bone was tougher than low Pyd/Dpd bone. Additionally, high Pyd/Dpd was less glycated, had more favorable microarchitecture, and contained less microdamage than low Pyd/Dpd bone. Our results reconfirm that enzymatic crosslinking in bone is a favorable process.

Our data should be considered in light of a limitation. 1–2 data points, associated with high amount of pentosidine, leverage the magnitude of association between pentosidine and mechanical properties (i.e. yield strain, Figure 2). Because removal of these points was not statistically justifiable, these points remained in our final analyses. A previous study on human cancellous bone also showed several donors with high pentosidine levels (beyond 40 mmol pentosidine per mol collagen) similar to the higher values seen in our work [50]. However, in order to further clarify the relationship between non-enzymatic glycation and mechanical properties, future work should consider inclusion of more samples with high pentosidine concentrations, associated with advanced age [39], diabetes [51]–[52] and anti-resorptive treatments [37].

In conclusion, we found that non-enzymatic glycation heterogeneously modified cancellous bone's trabecular microarchitecture where high amounts of AGEs were found in trabecular rods and with linear microcracks. We found that the extent of NEG in human tibial cancellous bone was the dominant predictor of bone fragility and was associated with changes in microdamage and microarchitecture.
